# Allometric relationships in morphological traits associated with foraging, swimming ability, and predator defense reveal adaptations toward brackish and freshwater environments in the threespine stickleback

**DOI:** 10.1002/ece3.6945

**Published:** 2020-10-29

**Authors:** Annette Taugbøl, Thomas P. Quinn, Kjartan Østbye, Leif Asbjørn Vøllestad

**Affiliations:** ^1^ Department of Bioscience Centre for Ecological and Evolutionary Synthesis (CEES) University of Oslo Blindern Norway; ^2^ Human Dimension Department Norwegian Institute for Nature Research (NINA) Lillehammer Norway; ^3^ School of Aquatic and Fishery Sciences University of Washington Seattle WA USA; ^4^ Faculty of Applied Ecology, Agricultural Sciences and Biotechnology Department of Forestry and Wildlife Management Inland Norway University of Applied Sciences Koppang Norway

**Keywords:** allometry, *Gasterosteus aculeatus*, lateral plates, morphology, spines

## Abstract

Freshwater colonization by threespine stickleback has led to divergence in morphology between ancestral marine and derived freshwater populations, making them ideal for studying natural selection on phenotypes. In an open brackish–freshwater system, we previously discovered two genetically distinct stickleback populations that also differ in geometric shape: one mainly found in the brackish water lagoon and one throughout the freshwater system. As shape and size are not perfectly correlated, the aim of this study was to identify the morphological trait(s) that separated the populations in geometric shape. We measured 23 phenotypes likely to be important for foraging, swimming capacity, and defense against predation. The lateral plate morphs in freshwater displayed few significant changes in trait sizes, but the low plated expressed feeding traits more associated with benthic habitats. When comparing the completely plated genetically assigned populations, the freshwater, the hybrids, the migrants and the lagoon fish, many of the linear traits had different slopes and intercepts in trait‐size regressions, precluding our ability to directly compare all traits simultaneously, which most likely results from low variation in body length for the lagoon and migrant population. We found the lagoon stickleback population to be more specialized toward the littoral zone, displaying benthic traits such as large, deep bodies with smaller eyes compared to the freshwater completely plated morph. Further, the lagoon and migrant fish had an overall higher body coverage of lateral plates compared to freshwater fish, and the dorsal and pelvic spines were longer. Evolutionary constraints due to allometric scaling relationships could explain the observed, overall restricted, differences in morphology between the sticklebacks in this study, as most traits have diversified in common allometric trajectories. The observed differences in foraging and antipredation traits between the fish with a lagoon and freshwater genetic signature are likely a result of genetic or plastic adaptations toward brackish and freshwater environments.

## Graphical abstract

By comparing 23 morphotypes on 215 stickleabacks, we found low plated morphs in freshwater and the completely plated lagoon fish to express a somewhat more benthic morphology.

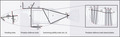

## 1 INTRODUCTION

When dispersing into new environments, novel resources, competitors, and predation regimes are agents of natural selection that may lead to new phenotypic optima (Schluter, 2000; Schluter & Conte, 2009). In general, the evolutionary and phenotypic response to such new selection regimes, or environmental conditions, will likely be contingent upon the genetic diversity of the founders, gene flow, and the evolvability of traits. For aquatic organisms, the transition from marine to freshwater habitats represents a considerable change in biotic and abiotic selective forces, and few species make this shift and retain populations in both environments. A fundamental question in evolutionary biology is how morphological traits are shaped and maintained in the environment. One way of studying environmental effects is hence to compare populations of the same species inhabiting marine to freshwater environments.

The threespine stickleback fish (*Gasterosteus aculeatus,* hereafter termed stickleback) is an attractive model organism for studying adaptation to marine and freshwater environments. The stickleback inhabits a wide range of salinities and varies extensively in morphology, both within and among populations (Klepaker, 1995; Lucek et al., 2010; McKinnon & Rundle, 2002). All present freshwater populations are believed to have descended from marine ancestors (Bell, 1981), and the numerous independent postglacial invasions of freshwater habitats have resulted in parallel evolution of a set of predictable phenotypes. The best known example of these changes in the stickleback is the repeated evolutionary loss of lateral plates in freshwater populations (Bell & Foster, 1994).

The shape and size of fishes are affected by both genetic (Arnegard et al., 2014) and plastic (environmental) factors (Day et al., 1994; Wimberger, 1992). Experimental studies indicate, for instance, that salinity alone can account for a large amount of the observed morphological shape differences between marine and freshwater stickleback (Mazzarella et al., 2015). Other studies indicate that food type and habitat complexity can also explain much of the morphological divergence, as stickleback feeding on benthic prey typically have smaller eyes, larger mouths, and deeper heads (McGee et al., 2013; McGee & Wainwright, 2013). The limnetic form, however, which preys on smaller organisms such as zooplankton, has a more streamlined body (Hart & Gill, 1994; McPhail, 1984, 1992). The differentiation along this benthic‐limnetic axis is usually continuous, but it sometimes results in populations or morphs of stickleback that feed almost exclusively on one or the other prey type (Gross & Anderson, 1984; Lavin & McPhail, 1986). Intermediate phenotypes tend to have reduced fitness, promoting ecological speciation (Nosil, 2012; Schluter, 1994).

The stickleback is small (<10 cm) and is preyed upon by a wide range of predators (Reimchen, 1994). As predation is a significant selective force that usually differs with salinity, it promotes multiple types of antipredator adaptions, including changes in morphology, behavior, and life history (Brönmark & Miner, 1992; Magurran, 1990). Having more bony lateral plates, as found in the marine stickleback, increases the probability of survival following an attack from a predatory fish (Hagen & Gilbertson, 1972; Reimchen, 1991, 1992). Therefore, a reduction of piscivore predation pressure in fresh water has been one of the hypotheses suggested to explain lateral plate reduction in freshwater stickleback. Freshwater populations are typically of the low‐plated morph, but completely plated stickleback do occur in fresh water (Hagen & Moodie, 1982; Kitano et al., 2008; Leinonen et al., 2012), either as resident individuals, or as anadromous fish breeding there (Harvey et al., 1997; McKinnon & Rundle, 2002; Taugbøl et al., 2014). The anterior plates are seemingly under strong selection, as few populations completely lack plates in the head region, but nonplated sticklebacks do exist (Deagle et al., 2013; Klepaker, 1995; Mazzarella et al., 2016). The anterior plates protect against puncturing injuries and also buttress the dorsal and pelvic spines, that, when erect, increase the effective size of the stickleback (Hoogland et al., 1957; Reimchen, 1983). The spines probably also function as a warning to gape‐limited piscivores (Hoogland et al., 1957). In areas where predators are common, the sticklebacks spines are often significantly longer than in areas where predators are absent or sparse (Hagen & Gilbertson, 1972; Zeller et al., 2012).

**Figure 1:**
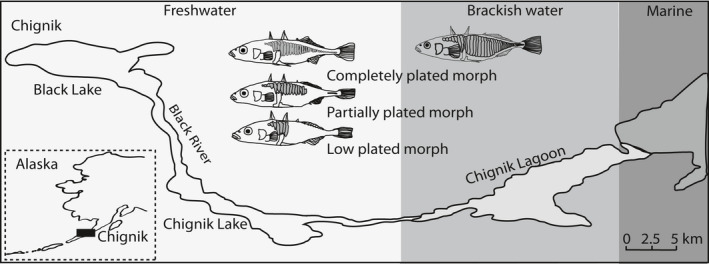
Study area and stickleback morphs. Inserted map of Alaska showing the position of the Chignik Lake system and locations of the four sampling sites as points within Chignik. The graded color is according to salinity: light gray being freshwater, gray being brackish water, and dark gray being marine salt water. The figure also illustrates the three stickleback morphs in the system, and where they were sampled. The completely plated morph has a full row of plates on both sides of the body, the partially plated have a gap in the row of plates, and the low plated has plates in the anterior region only, providing structural support for the pelvic‐ and dorsal spines. The illustrated fish are exemplified based on pictures of the morphs in the system

There are many documented examples of morphological evolution in freshwater populations after colonization by a marine ancestor, where the similar freshwater phenotypes are interpreted as a result of natural selection causing the populations to adapt to the new environmental conditions. Despite numerous studies, there is still no conclusive evidence for any major selective drivers of the phenotypic variations (i.e., shifts in diet, competition, predation, or a combination of these factors). Assuming that all freshwater lineages originated from the same ancestral population (Bell & Foster, 1994), it can be hypothesized that descendant freshwater populations inherited similar genetic architectures and developmental constraints and that there are allometric limitations to the evolvability of the traits. The stickleback included in this study is part of a subsample from a brackish water gradient in Chignik, southwestern Alaska (Figure 1), where we documented two genetically differentiated populations, one in brackish water and one in fresh water, with a few hybrids (Taugbøl et al., 2014). Individuals from the two genetically assigned populations also differed in size and geometric body shape. All individuals assigned to the brackish water population were completely plated (Figure 1), and some of these individuals were also found in fresh water as migrants. The individuals assigned to the freshwater population were either of the completely, partially, or low‐plated morph. None of the assigned freshwater individuals were found in brackish water. As size and shape are only imperfectly correlated, and their correlation is determined by the allometric relationships of the various body parts (Schlichting & Pigliucci, 1998), this paper aims to investigate allometric relationships in several ecologically important metric (linear) traits known to be important in the stickleback (Aguirre et al., 2008; Dalziel et al., 2012; Leinonen et al., 2006; Sharpe et al., 2008; Walker, 1997). We measured traits associated with feeding (head shape), swimming (body shape), and predator defense (size of dorsal and pelvic spines; size and width of lateral plates), to test for differences in linear traits in (a) completely, partially, and low‐plated fish genetically assigned to the freshwater population, and (b) in individuals assigned to the different genetic populations and their hybrids. We did this by using principal component analyses (PCA) to extract the most important traits that separated the groups and illustrated the differences with plots of allometric scaling relationships.

## 2 MATERIALS AND METHODS

### 2.1 Study area and fish community

Fish were collected from one site in each of four locations from the Chignik Lake system in southwestern Alaska (56°25'40"N, 158°75'60"W) (Figure 1); Chignik Lagoon, Chignik Lake, Black River and Black Lake. The Chignik Lagoon is an estuary ranging about 12 km from Chignik Bay up to the Chignik River. Depending on the location in the lagoon and the stage of the tide, the salinity ranges from 0 to about 30 ‰ (Simmons et al., 2013). Tidal amplitudes that exceed 3 m can expose half the estuarine substrate, largely covered by eelgrass (*Zostera spp*.). The Chignik River (7.2 km long) drains Chignik Lake (22 km^2^), a deep lake (maximum depth of 64 m) with a shoreline dominated by gravel. The Black River (12 km) connects Chignik Lake to Black Lake, which is larger (41 km^2^) but much shallower (maximum depth 4 m) than Chignik Lake. Black Lake rapidly warms in the spring and is highly productive with abundant vegetation and provides good breeding habitat for threespine stickleback (Narver, 1969). The fish communities of the two lakes are dominated numerically by threespine sticklebacks and juvenile sockeye salmon, *Oncorhynchus nerka* (Westley et al., 2010). The main potential fish predators in the lakes are juvenile coho salmon (*O. kisutch*) and Dolly Varden (*Salvelinus malma)* (Narver & Dahlberg, 1965; Roos, 1959; Ruggerone, 1992). The main fish predator in the lagoon is Dolly Varden. Analysis of the content of 3,000 stomachs of juvenile coho salmon from a survey in the 1990s found no stickleback (Ruggerone, 1992). Similar samples of Dolly Varden in the lagoon indicated that they consume primarily invertebrates such as amphipods, and when they eat fish it is mostly sand lance, *Ammodytes hexapterus* (Bond, 2013; Narver & Dahlberg, 1965; Roos, 1959). The system is also inhabited by freshwater sculpins *Cottus aleuticus* and *C. cognatus* (Quinn et al., 2012). Although few studies have investigated whether these sculpin species prey on stickleback, their congener, the prickly sculpin, *C. asper*, feed on sticklebacks (McPhail, 2007; Miller et al., 2015), and different sculpin species tend to have very similar diets (Brown et al., 1995). There are also a number of bird species in the area (Narver, 1970), many of which may prey on stickleback (Reimchen, 1988, 1994; Whoriskey & FitzGerald, 1985).

### 2.2 Stickleback collection

Adult threespine sticklebacks were collected using beach seines (35 × 4 m, 3 mm mesh), tow nets (1.8 × 2.7 m), and fyke nets (1.22 m^2^ frame with 3–5 m wings) during the two last weeks of June 2009. The sampling was done during the breeding season for sticklebacks. After collection, the fish were stored in 95% ethanol. We measured fork length to the nearest mm in the laboratory and discarded fish under 4 cm as all bony plates might not be fully developed until the fish reaches this size (Bell, 1981). Also, as the fish in this study were not aged, discarding individuals under 4 cm should leave only fish that was older than 1 year of age (Rollins, 2017; Wootton, 1976). All fish were stained in alizarin red using the modified protocol after Dingerkus and Uhler (1977), and all the lateral plates were counted directly on both sides and classified to morph according to Wootton (1976). After further discarding fish that had acquired an unnatural body curve due to storage and staining, the total sample sizes were 91 from Chignik Lagoon, 73 from Chignik Lake, 72 from the Black River, and 27 from Black Lake.

### 2.3 Categorization of fish

All the sampled fish were genotyped for 14 neutral microsatellites and a sex‐linked marker (see Taugbøl et al., 2014 for further details) that clearly separated the fish into two main genetic clusters; one comprised all the individuals sampled in the lagoon (hereafter called lagoon fish; *n* = 92), and one included fish sampled across the three freshwater sites (hereafter freshwater fish; *n* = 136). In addition, 33 individuals sampled in fresh water were genetically identified as belonging to the lagoon population (hereafter called migrants), and 11 individuals were identified as first‐generation hybrids between the two genetic populations. None of the fish sampled in the lagoon was genetically assigned to the freshwater population. We do not know whether the lagoon fish represent marine stickleback, or whether they have a different life history and constitute their own gene pool, as we do not have any samples from local oceanic stickleback. All fish from the lagoon, the migrants, and the hybrids were completely plated, whereas in fresh water, 55 were completely plated, 57 were partially plated, and 27 were low‐plated. As only 19% of the sampled individuals were males and morphological differences between sexes are known for stickleback (Aguirre et al., 2008; Kitano et al., 2007), we therefore only focus on the females (Table 1).

**Table 1:** Stickleback sorted in groups based on their genetic composition and morphology, the number of individuals in each group (N), length (in cm ± standard deviation, sd), and total plate numbers on the right side of the fish. The completely plated morphs in freshwater are included in both datasets

Group	*N*	Length ± sd	Plate numbers ± sd
Low plated	16	5.78 ± 0.71	6.87 ± 0.89
Partial plated	41	5.81 ± 0.69	26.64 ± 4.46
Completely plated/ Freshwater	35	5.71 ± 0.81	32.31 ± 0.82
Hybrids	8	7.13 ± 1.25	33.0 ± 1.22
Migrants	28	7.98 ± 0.40	33.5 ± 0.72
Lagoon	84	8.10 ± 0.39	33.60 ± 0.66

### 2.4 Morphological measures of metric traits

**Figure 2:**
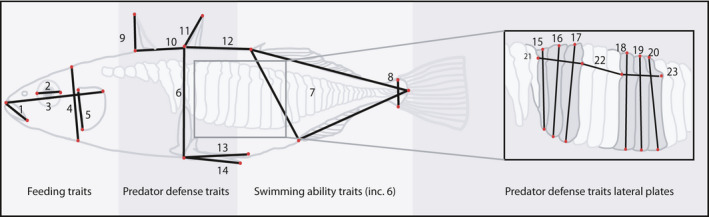
Linear measurements. Outline drawing of a threespine stickleback sampled from Chignik Lagoon illustrating distance measurements recorded from each individual with the help of digitalized landmarks (marked in red). The traits were separated into four groups: foraging, predator defense traits linked to spines, swimming ability traits, and lateral plate traits (measured on completely plated individuals only). More detailed information on the measured traits is found in the Methods

Four types of morphological traits were measured: (a) traits reflecting head shape and thus important for feeding; (b) spine traits important as defense against predators; (c) traits important for swimming/movement, and (d) lateral plate traits important as defense against predators. All fish were placed on a piece of clay to reduce bending and tilting and were photographed on the left side from directly above using a digital SLR‐camera (10 Mp) with a macro lens. A total of 37 landmarks were placed on the completely plated fish, whereas only 29 landmarks could be placed on the low‐ and partially plated fish (Figure 2) using tpsDIG2 (Rholf, 2005).

Information from the landmark configuration (Figure 2) was used to extract several linear measurements/traits for each fish: 15 traits for the low‐ and partially plated fish and 24 traits for the completely plated fish. All linear measurements were extracted using R version 3.6.1 (R Development Core Team, 2011). We extracted five traits from the head region for all fish (traits 1–5 in Figure 2a): mouth size (1), eye diameter (2), head length (3), head depth (4), and operculum size (depth) (5). To analyze for variation in traits potentially important for swimming performance (Dalziel et al., 2012), we extracted three traits (traits 6–8 in Figure 2a): body depth (6), caudal area (7), and peduncle width (8). We further extracted six traits related to the dorsal and pelvic spines from all fish (traits 9–14 in Figure 2a): length of spine one (9), distance between spines one and two (10), length of spine two (11), distance between spines two and three (12), length of the pelvic support structure (13), and length of the pelvic spine (14). For the completely plated fish, we also measured nine linear traits associated with the plates (traits 15–23, Figure 2b): the height of six plates, (traits 15–20) and the width of three combined plates (traits 21–23) (Figure 2b). We also calculated a proxy for plate coverage by dividing the length of each plate on body depth (trait 24, not illustrated).

### 2.5 Data analysis

All statistical analyses were done in R (R Development Core Team, 2011). The first dorsal spine (trait 9) was excluded from all analyses as the tip of the spine often was submerged in the clay material that kept the fish stable, and the pelvic spine (trait 14) was also excluded as it was challenging to place the landmark correctly. This left 13 traits that could be compared for all fish, and an additional 9 traits that could be compared for the completely plated fish only (see Figure 2 for details on the traits). We tested for differences between lateral plated morphs in the freshwater population; between the lagoon and freshwater completely plated; and between completely plated fish genetically assigned to either population or their hybrids in three separate analysis (Table 1). As the low‐plated morph differed slightly in the freshwater fish comparison, we only included the completely plated morphs in the freshwater and lagoon comparison.

Since evolutionary allometry is the log–log regression of the mean trait size on mean body length across populations, we used standardized major axis regression (Warton et al., 2006) on all traits between the different groups as a first step to test for differences in allometric scaling relationships (slope and intercept). More specifically, we used the functions “sma” and “ma” in R package “smart” (Warton et al., 2012). When the slope and/or the intercept of a trait differed significantly, we determined which groups differed from each other by mean‐centering the log body length of the fish around zero to make the intercept in the model equal to the trait mean within each treatment (mean of zero, sd = 1). This standardization enabled us to estimate the proportional trait change across morph type and genetic population as the ratio between the intercepts by using a general linear model (GLM): trait ~ bodylength × morph.

To compare between traits and groups, each trait was size‐corrected by expressing it as residuals from ordinary least‐squares regression on body size on logged values or on body depth for the lateral plate lengths (trait 15–20, Figure 2). We extracted the residuals using all individuals in the specific comparisons and checked the normality distribution of the data with qq‐plots. To determine morphological traits that best describes intergroup differences, we applied principal component analysis (PCA) on the residual data, both grouped into feeding traits, predator defense traits and swimming ability traits, and on all traits combined. The approach of using size‐corrected measures instead of defining size as the first principle component is strongly advocated when comparing several groups (Berner, 2011), as the orientation of the first principal component (PC1) also influences the orientation of the remaining PC’s. As PCA provides a multivariate description of allometry for a single group, despite including data pooled from several groups, this can lead to wrong interpretations (McCoy et al., 2006), and we therefore also ran PCA analysis for each group, and each group and trait separately, and checked against the common PCA’s. The resulting comparisons gave similar outcomes, and we hence only present data from the pooled PCA’s. The PCA’s were performed on the covariance matrix of the residuals in R by the use of two packages: FactoMiner for the analysis (Le et al., 2008) and factoextra (Kassambara & Mundt, 2020) for visualization. By using the function PCA() in FactoMiner, the program standardizes the values automatically by scaling the data to unit variance, making the variables more comparable. We screened the variables total eigenvalues and contributions and plotted the individual coordinate values for PC1 and PC2 as these are the most important dimensions in explaining the variability, in addition to the five variables with the highest contributions as arrows. The contributions of each variable were calculated as follows: (variable.cos2*100/total cos2 of the component), where cos2 represents the quality of the representation for the variables on the factor map, as integrated in FactoMiner (Le et al., 2008). By the use of a scree plot of the percentage of explained variance by each dimension, we kept the PC’s that explained most of the variability for further analysis, where we extracted the coordinate values and tested each separately for differences within morph or population using GLM; PCaxis ~ morph or population.

## 3 RESULTS

### 3.1 Comparing the different morphs genetically assigned to the freshwater population

**Figure 3:**
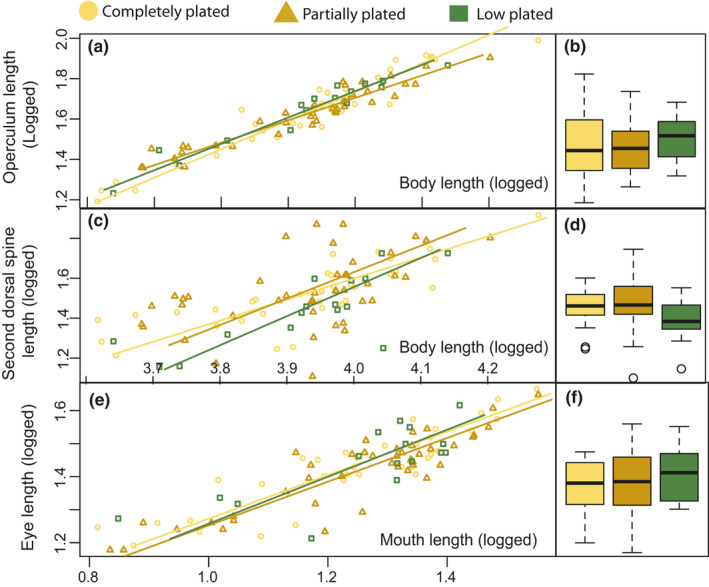
Standardized major axis regression. Allometric relationships for the freshwater morphs for 3 of the 13 investigated linear traits and their residuals from common slopes in a GLM as used in the principle component analysis (common slope not shown); a) operculum length on body length, b) operculum residuals, c) second dorsal spine on body length, second dorsal spine residuals, e) eye length on mouth length, and f) mouth length residuals. All data are logged. Completely plated is plotted in yellow circles, partially plated is plotted in brown triangles, and low‐plated individuals are plotted in green squares

The three morphs did not differ in body length (*F*_2, 91_ = 0.104, *p* = .901, Table 1). All linear traits were significantly correlated with length, and length alone could hence explain between 27.8% (second dorsal spine, partially plated) and 99.1% (caudal area, completely plated) of the trait variation. We tested for differences in allometric scaling relationships for all traits (Table S1), where we documented differences in the operculum slopes only (likelihood ratio tests _2_ = 8.11, *p* = .02). When testing operculum length separately on mean‐centered data in a GLM, we found that the partially plated individuals differed from the completely plated by having a significant interaction between operculum and body length (T = −2.90, *p* = .004, Figure 3a).

**Figure 4:**
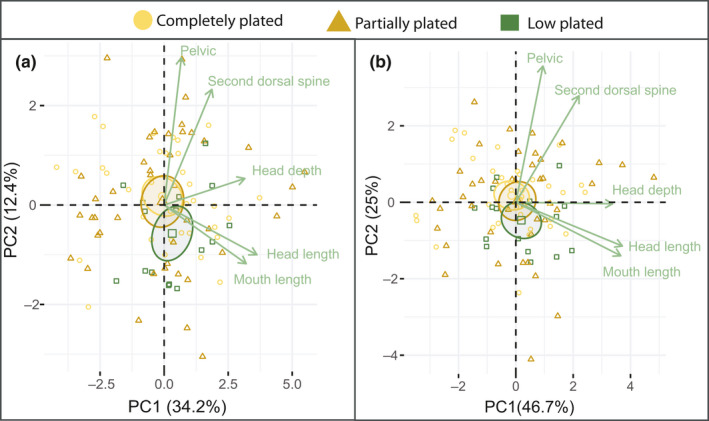
PCA for freshwater morphs. a) illustrates PC1 plotted against PC2 for all traits, and b) illustrates PC1 plotted against PC2 when only including the traits with the highest contribution from a). Both plots include the five most influential variables in green. Completely plated is plotted in yellow circles, partially plated is plotted in brown triangles, and low‐plated individuals are plotted in green squares

There were no major significant differences between morphs when testing the morphological trait groups separately with PCA on the residual data, but the low‐plated individuals were significantly different in PC3 (*F*_2, 91_ = 3.093, *p* = .05) when comparing predator defense traits, having on average a shorter second dorsal spine (Figure 3c). When testing all 12 traits together in a PCA, the first PC explained 30.6%, PC2 explained 15.6%, and PC3 explained 10.6% of the variation, a total of 56.8% for the three first PCAs. PC1 was mostly related to head and mouth length, (Table 2; Figure 3e), and the main contributors to PC2 were the two lengths between the dorsal spines (trait 10 and 12). As these variables were highly correlated (94,6%, *p* = <0.001), we removed trait 12 and reran the analysis (Table 2; Figure 4a). PC1 now explained 34.2% and PC2 12.4%. The low‐plated individuals were significantly different from the completely plated in PC2 (Figure 4a T = −2.14, *p* = .035), indicating that the low‐plated morphs had longer mouths and heads and shorter dorsal spines (Figure 3b, d and f). The five variables with the highest contributions to PC1 and PC2 were mouth length, head length, head depth, the length of the second dorsal spine, and the length of the pelvic (Figure 4b). By reanalyzing the data using these five variables, we could explain a total of 71,7% of the variation by the two first PC’s, the low‐plated morphs being marginally significantly different from the completely plated individuals (Figure 4b, T = −1.93, *p* = .056).

**Table 2:** Coordinates for the variables for the freshwater morph dataset, the lagoon and freshwater dataset and the lagoon, migrant, hybrids, and freshwater dataset

	Freshwater morphs			Lagoon and freshwater completely plated			Lagoon, migrants, hybrids, and freshwater		
	PC1	PC2	PC3	PC1	PC2	PC3	PC1	PC2	PC3
Mouth length	0.7557	−0.0896	−0.1332	0.0037	−0.0616	0.0243	0.0194	0.0630	−0.0328
Eye length	0.6426	−0.1577	0.0747	0.0037	−0.0383	0.0185	0.0018	0.0398	−0.0080
Head length	0.8536	−0.0585	−0.1305	−0.0019	−0.0375	0.0213	0.0024	0.0429	−0.0135
Head depth	0.7364	0.1188	−0.0103	0.0068	−0.0132	0.0195	0.0081	0.0238	−0.0024
Operculum	0.5724	0.1447	−0.0599	0.0030	−0.0119	0.0364	0.0057	0.0302	0.0117
Body depth	0.4180	0.3162	−0.2214	−0.0269	0.0025	0.0052	−0.0358	0.0004	0.0047
Caudal area	−0.5760	−0.4197	0.3564	0.0015	0.0196	−0.0097	0.0022	−0.0181	0.0051
Peduncle width	0.5973	0.0695	0.1306	0.0023	−0.0041	0.0130	0.0070	0.0139	0.0062
Distance spine 1 and 2	−0.2941	0.7922	0.0524	0.0097	0.0157	0.0035	0.0099	−0.0121	0.0056
Second dorsal spine	0.4328	0.1697	0.6348	0.0348	0.0250	0.0093	0.0224	−0.0314	0.0018
Distance spine 2 nd 3	−0.2931	0.8440	−0.1766	−0.0011	0.0212	−0.0122	−0.0012	−0.0253	0.0062
Pelvic	0.1474	0.2729	0.7783	0.0274	0.0590	0.0627	0.0251	−0.0118	0.0693
Plate 1	‐	‐	‐	0.0420	0.0155	0.0589	0.0464	0.0530	0.0800
Plate 2	‐	‐	‐	0.0552	−0.0030	0.0205	0.0703	0.0243	0.0215
Plate 3	‐	‐	‐	0.0771	−0.0041	0.0100	0.0893	0.0068	0.0063
Plate 4	‐	‐	‐	0.1088	−0.0066	−0.0117	0.1235	−0.0115	−0.0131
Plate 5	‐	‐	‐	0.1178	−0.0070	−0.0187	0.1307	−0.0147	−0.0170
Plate 6	‐	‐	‐	0.1176	−0.0029	−0.0259	0.1296	−0.0216	−0.0197
Width 1	‐	‐	‐	−0.0006	0.0353	−0.0227	−0.0065	−0.0313	0.0184
Width 2	‐	‐	‐	−0.0140	0.0387	0.0067	−0.0100	−0.0273	0.0260
Width 3	‐	‐	‐	0.0061	0.0214	−0.0166	−0.0053	−0.0332	0.0173

### 3.2 Comparing freshwater and lagoon fish

**Figure 5:**
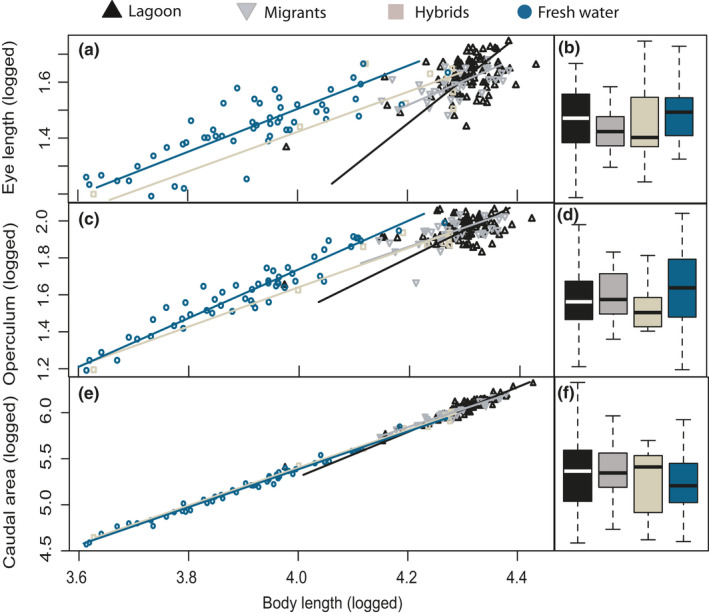
Standardized major axis regression. Allometric relationships for the lagoon, migrants, hybrids, and freshwater completely plated fish for 3 of the 21 investigated linear traits and their residuals from common slopes in a GLM as used in the principle component analysis (common slope not shown). a) Eye length on body length, b) eye length residuals, c) operculum on body length, operculum residuals, e) caudal area on body length and f) caudal area residuals. All data are logged. The lagoon fish are plotted in black triangles, the migrants in gray triangles, the hybrids in light brown squares, and the fresh water in blue circles. The plots are made in R with the use of the smatR package

When comparing the four groups of completely plated fish (the lagoon, migrants, hybrids, and completely plated freshwater fish, Table 1), most traits differed significantly in slope and elevation, or separately in slope or elevation (Figure 5a and c; Table S2). We therefore tested for differences between the groups for each trait separately by standardizing the body length and used linear models for the evolutionary allometry parameter estimates. The results of the mean log eigenvalues for each genetic population are given in Table 2, where we report the proportional differences between the freshwater and the lagoon fish only and we therefore report the findings here. The freshwater fish had overall larger values in the head region, with an overall larger size (length and width), and a larger mouth (6,7%), eye radius (5.1%; Figure 5a), and operculum (6.3%) in proportion to the body compared to the lagoon fish. The swimming ability traits were more equal in size (Table 2); freshwater fish had larger peduncle width (5.3%). Caudal area had the highest coefficient of determination (R^2^ = 99.01%; Figure 5c). The largest proportional trait change between the lagoon and freshwater population was the width of the three first measured plates (trait 21), which was on average 8.1% longer in the lagoon population (Table 2; Figure 8d). However, all of these results should be interpreted with caution, as the lagoon fish varied much less in body length than did the freshwater sample.

**Figure 6:**
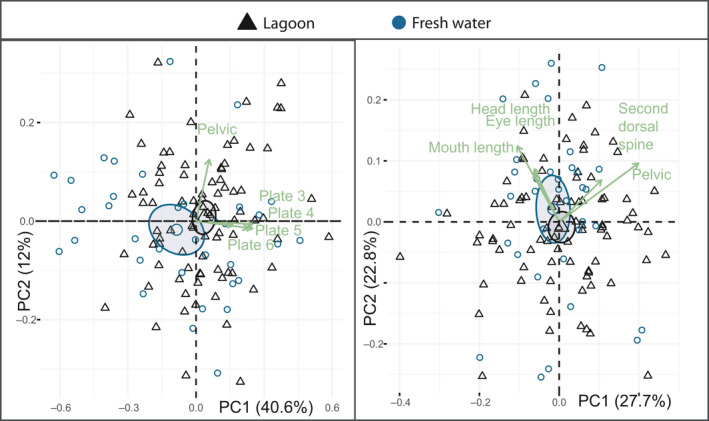
PCA for lagoon and freshwater completely plated fish. a) illustrates PC1 plotted against PC2 for all traits, and b) illustrates PC1 plotted against PC2 when excluding the lateral plate traits. Both plots include the five most influential variables in green. The lagoon fish are plotted in black triangles and the fresh water in blue circles

There were no major differences in PC1, PC2, or PC3 when comparing the freshwater and lagoon population's morphological trait groups separately with PCA, except for the lateral plate comparison. When only including the lateral plate traits, trait 15:23, PC1 explained 63.2% of the variation that was especially linked to plate 4–6, and the lagoon population differed significantly from the freshwater population by having on average 0.12 larger values for PC1 (T = 2.75, *p* = .01; data not shown). When testing all traits for the completely plated lagoon and freshwater common residuals, PC1, PC2, and PC3 explained 40.6%, 12.0%, and 10.3% of the total variation, respectively. The lagoon and freshwater fish were significantly different in PC1; the lagoon fish had on average 0.12 larger proportional trait values compared to freshwater fish (T=−2.65, *p* = .01), indicating larger plates and a longer pelvic spine (Figure 6a). As the plate traits were highly correlated, we also ran the analysis without the plate traits, resulting in a lower separation between the fresh and lagoon population, indicating larger values for the freshwater population in the head (Figure 6b).

### 3.3 Comparing completely plated freshwater, migrants, hybrids, and lagoon fish

**Figure 7:**
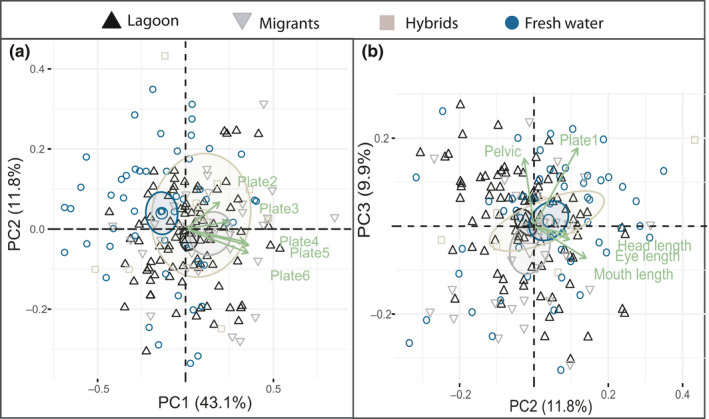
PCA for the lagoon, the migrants, the hybrids, and the freshwater population. a) illustrates PC1 plotted against PC2 for all traits, and b)illustrates PC2 plotted against PC3 for all traits. Both plots include the five most influential variables in green. The lagoon fish are plotted in black triangles, the migrants in gray triangles, the hybrids in light brown squares, and the fresh water in blue circles

**Figure 8:**
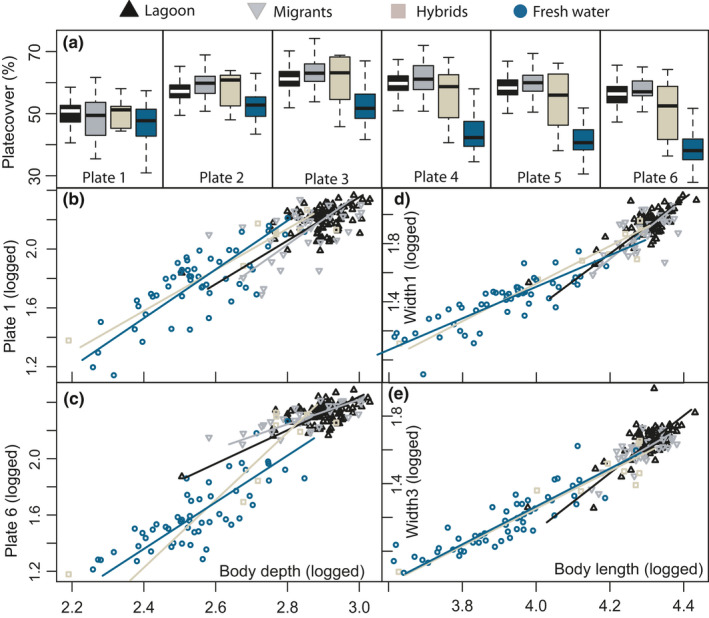
Lateral plates. a) The figure is illustrating the percent body cover for plate 1‐plate 6 for the lagoon fish (black), the migrants (gray), the hybrids (light brown), and the freshwater fish (blue). The boxplots are illustrating the 25%–75% quantiles (boxes), median (black horizontal line), 95% limits (bars), and outliers (open circles). b) Allometric relationships for plate1 on body depth, c) allometric relationship for plate6 on body depth, d) allometric relationship for the width of the first three measured plates(width1) on body length, and e) allometric relationship for the width of the last three measured plates (width3) on body length. All data are logged

As we have reported on the main findings between the lagoon and freshwater populations in the previous section, we will only report main finding between the other groups here. When analyzing the head traits with PCA, the migrants were significantly different in PC3 (T=−2.98, *p* = .003), indicating that they had smaller eyes and narrower heads compared to the lagoon fish. When analyzing swimming ability traits, the migrants differed from the lagoon in both PC1 and PC2 indicating a narrower body (T = −0.02, *p* = .05; T = −0.02, *p* = .02, respectively). The hybrids had significantly smaller values for PC1 and PC2 when comparing the spine lengths (T = −0.01 *p* = .02; T = −0.1, *p* = .05, respectively), where the second dorsal spine had the highest contribution to the PC’s. PC1 explained 64% when comparing the lateral plate traits, and the migrants were positively significantly different from the lagoon, having on average 0.12 larger plate sizes (T = 2.48, *p* = .01). When including all traits in the analysis, the plate traits (traits 15–20) were the measurements with the highest contributions to PC1, eye length, operculum and length of pelvic to PC2, and mouth length, pelvic, and length of plate1 for PC3 (Figure 7a, b). When excluding the plate traits from the analysis, mouth length, eye length, head length, pelvic spine, and second dorsal spine contributed the most. The different lateral plates covered from an average of 63.9% (plate 6 in freshwater) to 83.7% (plate 3 in migrants) of the body depth. Overall, the plates covered more of the body in the lagoon and the migrant individuals, when compared to the hybrids and freshwater fish (Figure 8; Table 3).

**Table 3:** Allometric parameter estimates for the completely plated groups

Linear trait		Mean log trait size				Proportional mean trait change
		Lag	Mig	Hyb	Fresh	
Mouth length		1.49	1.54	1.53	1.59	−6.7*
eye diameter		1.57	1.55	1.56	1.65	−5.1**
Operculum		1.91	1.88	1.86	2.03	−6.3***
Head length		2.87	2.87	2.89	2.96	−3.1***
Head depth		2.5	2.45	2.47	2.52	−0.8
Body depth		2.84	2.76	2.8	2.84	‐
Caudal area		5.87	5.9	5.86	5.82	0.8*
Peduncle width		1.12	1.11	1.12	1.18	−5.3*
Pelvic		2.45	2.4	2.4	2.43	0.8
Second dorsal spine		1.9	1.82	1.77	1.79	6.1*
Distance spine 1 and 2		2.12	2.13	2.11	2.09	1.4
Distance spine 2 and 3		2.94	2.95	2.93	2.87	2.4***
Plate 1		2.19	2.03	2.11	2.16	1.3
Plate 2		2.31	2.28	2.27	2.28	1.3
Plate 3		2.37	2.33	2.32	2.31	2.5
Plate 4		2.35	2.29	2.27	2.22	5.8**
Plate 5		2.33	2.25	2.24	2.16	7.8***
Plate 6		2.28	2.2	2.2	2.12	7.5***
Width 1		1.85	1.79	1.79	1.71	8.1***
Width 2		1.68	1.65	1.65	1.64	2.4
Width 3	1.58		1.55	1.49	1.52	3.4

Note

Mean log trait size refers to the intercept of the model, proportional mean trait change is calculated as the ratio between the two trait means between the lagoon and freshwater individuals, where positive numbers indicate the lagoon fish to have larger values compared to the freshwater fish, *indicate significance; *p < .05, **p < .01, ***p < .001, allometric slope represents the intercept on all populations combined, SE; standard error.

## 4 DISCUSSION

The focus of the present study was to test whether morphological differences between stickleback populations in an open system could be linked to differences in functional traits important for foraging, swimming, and defense against predation. When comparing the three lateral plate morphs within the freshwater population, the low‐plated morph had a longer head, a larger mouth, and a shorter second dorsal spine. When comparing the four groups of completely plated fish, most traits for foraging, swimming capacity, and plate coverage had different allometric slopes and/or intercepts, implying that selection processes and/or plasticity are dividing the groups. Conversely, most traits measured for predator defense followed common allometric trajectories, likely resulting from shared evolutionary history and constraints in evolvability of the traits. The lagoon fish and migrants had overall smaller values in the head and larger values in the antipredation traits. This was especially evident in the lateral plate size and coverage of the body, where the freshwater fish had a reduced armor coverage, likely from selection and genetic regulation.

Freshwater systems containing all three lateral plated morphs are not very common. Sympatric lateral plate morphs have been found to vary little in neutral genetic differences (Østbye et al., 2018; Pedersen et al., 2017) and have seldom been studied with respect to adaptations to foraging, swimming ability, and predator defense. Our results illustrate that most traits vary little between the lateral plate morphs in freshwater, but the low‐ and completely plated morphs were most different, and the partially plated morph was similar to the completely plated morph. The low‐plated morph had somewhat larger mouths and longer heads. This could indicate a larger maximum gape, which again could indicate selection on feeding performance on larger prey for the low‐plated morph. Low‐plated morphs from a brackish water lake in Norway fed more efficiently on larger benthic prey than the two other morphs (Bjærke et al., 2010), implying that the low‐plated morph in Chignik likely has a more benthic lifestyle and therefore is more adapted to freshwater habitats.

Operculum length was the only trait that had a significant different slope and elevation for the three lateral plates in freshwater, indicating a degree of ecotypic adaptation for the partially plated morph linked to size. When comparing the operculum sizes for the completely plated genetic groups (Figure 5c,d), the freshwater fish were larger, opposite to what we would expect, as other studies have found the operculum to be smaller in freshwater populations compared to anadromous ones (Kimmel et al., 2005). The size of the operculum varies between stickleback populations (Arif et al., 2009; Jamniczky et al., 2014; Kimmel et al., 2005); the larger operculum in freshwater fish in this system could indicate a more active pelagic lifestyle as more water can pass through the gills, but a larger size could also imply suction feeding ability for benthic prey (Day et al., 2015). Further, other studies have also found the ontogenetic growth of the operculum size and shape to have different allometric slopes and developmental endpoints, and the freshwater fish did not develop the full ancestral adult bone shape (Kimmel et al., 2012). This could be linked to osmoregulation, as young adult sticklebacks would continue their operculum bone development to the adult stage in salt water. Also, the skin inside the operculum in freshwater acclimated killifish (*Fundulus heteroclitus*) predominately contained chloride cells (Karnaky & Kinter, 1977), further indicating that the size differences in this study could be linked to osmoregulatory functions.

Despite being small, the observed morphological differences can have large effects in relative fitness for individuals (Parsons et al., 2011). Morphological measurements from the head indicated that the lagoon stickleback, with smaller eyes, shorter mouths, and shorter and broader heads, had somewhat more benthic features compared to the freshwater completely plated fish (Willacker et al., 2010). This is somewhat contrary to expectations, as marine sticklebacks tend to feed on planktonic prey, as inferred from gut content and gill‐raker analyses (Hart & Gill, 1994; Wootton, 1976). This foraging ecology is maintained in so‐called “limnetic” freshwater populations of stickleback in large, oligotrophic lakes (McPhail, 1984; Schluter, 1993; Walker, 1997). However, the habitat in Chignik Lagoon consists largely of seagrass, increasing the habitat complexity and probably also the likelihood of benthic feeding, thereby potentially selecting for a more benthic lifestyle. Further, other studies, focusing on Atlantic stickleback, also found that specimens from the marine environment had smaller heads and eyes compared to freshwater fish (Leinonen et al., 2006; Voje et al., 2013). The size of the eye also seems to be a very plastic trait in fish (Howland et al., 2004); the size might to some extent be related to predatory regimes, where different high‐predator scenarios are selecting for decreasing eye size or eye pigments (Frommen et al., 2011; Löennstedt et al., 2013; Zaret & Kerfoot, 1975), or high predatory regimes can also lead to increasing eye size (Ab Ghani et al., 2016; Miller et al., 2015). Further, larger eyes lead to improved visual sensitivity and resolution (Hairston et al., 1982) and could improve benthic feeding that is commonly associated with reduced light in the deeper parts of the lakes (Rick et al., 2012; Willacker et al., 2010). Linear allometric relationships between populations might result from genetic constraints in the founding population (Gould, 1971). The lagoon stickleback had less variation in body length and were also larger than the freshwater stickleback. McGugian (2010) found that the trait correlating the least with body length was eye diameter and head length, as bigger fish had smaller and shorter heads in comparison to their size, and Wund (2012) also found deeper bodies to correlate with a smaller eye size. These effects can be due to allometric constraints, as larger fish have allometrically smaller heads (McGuigan et al., 2010; Walker, 1997), which could partly explain the results also for these populations, as most of the fitted lines for the head‐measurements are close to the common trajectories, narrowing down the possible directions of evolution in trait space.

The trait with the smallest variation between individuals, giving the tightest fit between populations in the shared common allometric trajectory, was the caudal area (Figure 5e). This similarity is likely a result of swimming performance being important for all habitats and that the trait is under strong selection. Overall, the traits measured for swimming ability were not very different between the completely plated groups, as would have been expected, especially for the anadromous group that swim over 25 Km to breed. Locomotor performance is important for a wide range of ecological processes, such as foraging, courtship, and predator avoidance (Videler, 1993). Having a smaller and more bulky body size may increase foraging performance within dense vegetation (Stoner, 1982; Webb, 1984), as is found in the lagoon, whereas a large, slim body size decreases the cost of movement and is more suited in open habitats (Webb, 1984). The migrants did have reduced body depth and peduncle width, which could be a result of selection, or more likely, from energy deficiency after a long up‐river migration or a combination of these two factors.

The plate coverage was highest in the lagoon and migrant sticklebacks. This was consistent for all the measured traits, but especially for the sixth measured plate (Trait 20), where the plate covered on average 80.1% compared to 63.9% in the freshwater completely plated fish (Figure 8a). The difference suggests genetic regulation, likely from the gene GDF6 (growth differentiation factor 6), that has been linked to increased expression in freshwater fish, resulting in smaller plates (Indjeian et al., 2016). Smaller plates in freshwater might be beneficial in a number of ways, including faster burst swimming speeds (Bergström, 2002), maintenance of neutral buoyancy (Myhre & Klepaker, 2009) and metabolic demands (Grøtan et al., 2012). The plate number itself is also under strong selection. A typical trend in the stickleback freshwater invasion is that the number of bony lateral plates on both sides is reduced within few generations (Bell et al., 2004; Klepaker, 1993; Le Rouzic et al., 2011), creating a morph distribution that usually correspond to salinity, as the completely plated, the partially and the low‐plated morphs associate most commonly with high, intermediate, and low salinity, respectively. The “textbook example” of a stickleback hybrid zone further includes low‐plated morphs in freshwater, partially plated in the hybrid zone, and completely plated marine/anadromous fish (Bell & Foster, 1994; Hagen, 1967). The population structure between marine and freshwater stickleback in the present paper differs from most other stickleback gradients in that there is no evident hybrid zone and few identified hybrids, clearly indicating different roles of natural selection, pre‐, or postzygotic barriers between the populations. Further, the freshwater stickleback population in Chignik consists of all the three lateral plate morphs, with about 45% of the freshwater stickleback being completely plated and only 17% constituting low‐plated individuals, a fraction which have been similar at least since the 1960s (Narver, 1966, 1969) and they also constitute a genetic population different from the lagoon fish (Taugbøl et al., 2014). Fish predation could in theory explain the dominance of completely plated fish in the system, but the main potential fish predators in the system, Dolly Varden and coho salmon, seem to rarely prey on sticklebacks (Bond, 2013; Roos, 1959; Ruggerone, 1992) and with the high abundance of stickleback in the system (Harvey et al., 1997) the overall predation pressure is likely very low. Further, most lakes across the northern hemisphere have trout or other predatory fish present (Reimchen, 1994), and one would therefore expect completely plated sticklebacks in freshwater to be more common if predation were the single explanation for evolutionary plate loss in these fish. The evolutionary loss of plates is also accompanied by a change in the lateral line sensory system (Mills et al., 2014; Wark et al., 2012; Wark & Peichel, 2010), suggesting that the loss of plates might be due to selection on the lateral line rather than the plates. It is thus still uncertain which selective agent(s) account for the high degree of completely plated stickleback in the freshwater system in Chignik.

In conclusion, much of the morphological variation in stickleback populations is hypothesized to be related to foraging opportunities. The Chignik system consists of a deep lake and a shallow lake. We found the lagoon stickleback population to be more specialized toward the littoral zone, displaying benthic traits such as large, deep bodies with smaller eyes compared to the freshwater completely plated morph. The lagoon and migrant fish had larger lateral plates that covered more of the body, which was especially evident for the sixth measured plate (the plate roughly above the start of the anal fin). As the freshwater population has had had a stable lateral plate morph distribution since 1960s, it seems to be a selection pressure in freshwater that sustain the completely plated morph, and at the same time selects for smaller plates. When comparing the freshwater fish divided in three lateral plated morphs, the low‐plated morph expressed values more consistent with benthic feeding and smaller antipredation traits compared to the partial and completely plated morphs, likely a plastic response to selection on habitat preference.

## CONFLICT OF INTEREST

The authors declare no conflict of interest.

## AUTHOR CONTRIBUTION

**Annette Taugbøl:** Conceptualization (equal); Formal analysis (lead); Methodology (lead); Writing‐original draft (lead); Writing‐review & editing (equal). **Thomas Quinn:** Conceptualization (equal); Funding acquisition (equal); Resources (equal); Writing‐review & editing (equal). **Kjartan Østbye:** Conceptualization (equal); Writing‐review & editing (equal). **Leif Asbjørn Vøllestad:** Conceptualization (equal); Funding acquisition (equal); Project administration (equal); Supervision (equal); Writing‐review & editing (equal).

## Supporting information

Appendix S1

Appendix S2

Appendix S3

## Data Availability

All the relevant data are within the paper and in the Supporting Information file 3.
